# Temporal trend of sexual violence: time series analysis, Brazil, 2016–2022

**DOI:** 10.1590/S2237-96222026v35e20250416.en

**Published:** 2026-05-01

**Authors:** João Vitor Lima Pereira, Ludiane Rodrigues Dias Silva, Eduarda da Silva Miranda, Brenda Rainara Pereira da Silva, Izabel Cristina da Silva Carvalho, José Cláudio Garcia Lira, Adriene da Fonseca Rocha, Jéssica de Menezes Nogueira, Jardeliny Corrêa da Penha

**Affiliations:** 1 Universidade de Brasília, Programa de Residência Multiprofissional em Vigilância em Saúde, Brasília, DF, Brazil; 2 Universidade Federal do Piauí, Programa de Pós-Graduação em Saúde e Comunidade, Teresina, PI, Brazil; 3 Universidade Federal do Piauí, Coordenação do Curso de Bacharelado em Enfermagem, Floriano, PI, Brazil; 4 Universidade Federal do Piauí, Coordenação do Curso de Ciências Biológicas, Floriano, PI, Brazil

**Keywords:** Violence, Sexual Offenses, Brazil, Epidemiological Monitoring, Time Series Analysis, Violencia, Delitos Sexuales, Brasil, Monitoreo Epidemiológico, Análisis de Series Temporales

## Abstract

**Objective:**

To analyze the temporal trend of sexual violence in Brazil between 2016 and 2022.

**Methods:**

Time series study using reported cases of sexual violence between 2016 and 2022 from the Notifiable Diseases Information System (SINAN). The annual percent change (APC) and its corresponding 95% confidence interval were calculated using the Prais-Winsten generalized linear regression model.

**Results:**

A total of 299,962 cases of sexual violence were reported. An increasing trend was also observed at the national level (APC 8.13; 95%CI 3.38; 13.12; p-value 0.007) and in all geographic regions of Brazil: Central-West (APC 8.56; 95%CI 3.33; 14.07; p-value 0.008); Northeast (APC 10.61; 95%CI 6.60; 14.78; p-value 0.001); North (APC 5.06; 95%CI 1.09; 9.19; p-value 0.022); Southeast (APC 8.72; 95%CI 3.38; 14.36; p-value 0.008); and South (APC 7.01; 95%CI 0.41; 14.05; p-value 0.041). Regarding age group, sex, race/skin color, and education level of the victim, only four variables showed a stationary trend: age: ignored/blank (APC 25.90; 95%CI -34.38; 141.59; p-value 0.405), sex: male (APC 5.54; 95%CI -0.43; 11.88; p-value 0.063), race/skin color ignored/blank (APC -1.99; 95%CI -4.91; 1.01; p-value 0.147), and education level: illiterate (APC 1.68; 95%CI -2.81; 5.71; p-value 0.318). All other variables showed an increasing trend.

**Conclusion:**

The trend of sexual violence in Brazil remained upward, with a wide geographic and sociodemographic distribution.

## Resumen

**Objetivo:**

Analizar la tendencia temporal de la violencia sexual en Brasil entre 2016 y 2022.

**Métodos:**

Estudio de series temporales en el cual se utilizaron los casos de violencia sexual notificados, entre 2016 y 2022, en el Sistema de Información de Agravamientos de Notificación. Se calculó la Variación Porcentual Anual (VPA) y su respectivo Intervalo de Confianza del 95%, utilizando el modelo de análisis lineal generalizado de Prais-Winsten.

**Resultados:**

Se notificaron 299.962 casos de violencia sexual. También se observó una tendencia creciente a nivel nacional (VPA 8,13; IC95% 3,38; 13,12; valor de p 0,007) y en todas las regiones geográficas brasileñas: Centro-Oeste (VPA 8,56; IC95% 3,33; 14,07; valor de p 0,008); Nordeste (VPA 10,61; IC95% 6,60; 14,78; valor de p 0,001); Norte (VPA 5,06; IC95% 1,09; 9,19; valor de p 0,022); Sudeste (VPA 8,72; IC95% 3,38; 14,36; valor de p 0,008); y Sur (VPA 7,01; IC95% 0,41; 14,05; valor de p 0,041). Con respecto al grupo etario, sexo, raza/color de piel y escolaridad de la víctima, solo cuatro variables presentaron tendencia estacionaria: edad ignorada/en blanco (VPA 25,90; IC95% -34,38; 141,59; valor de p 0,405), sexo masculino (VPA 5,54; IC95% -0,43; 11,88; valor de p 0,063), raza/color de piel ignorada/en blanco (VPA -1,99; IC95% -4,91; 1,01; valor de p 0,147) y escolaridad analfabeta (VPA 1,68; IC95% -2,81; 5,71; valor de p 0,318). Las demás variables presentaron tendencias crecientes.

**Conclusión:**

La tendencia de la violencia sexual en Brasil se mantuvo en ascenso, con amplia distribución geográfica y sociodemográfica.

**Ethical aspects**

This research used public domain anonymized databases.

## Introduction 

Sexual violence can be understood as any action related to a person’s sexuality through coercion, including completed acts, attempts, or other forced practices (1). It can generate diverse impacts with repercussions at different points in the life and health of those who suffer from it, such as emotional implications that directly affect interpersonal, emotional, and sexual relationships, as well as family, social, and professional relationships. These consequences may stem from loss of trust in others, insecurity, or changes in the way victims perceive others and themselves, as they carry subjectively lived scars (2).

Worldwide, sexual violence is highly prevalent among women. In 2018, the World Health Organization (WHO) estimated that approximately 852 million women aged 15 to 49 had already experienced some form of physical and/or sexual violence perpetrated by intimate partners at some point in their lives, representing a major global public health challenge and a serious violation of human rights (3,4). In Brazil, regarding cases reported to police authorities through the Disque 100 (“Call 100”) hotline in 2023, there was an increase in reports of sexual violence, mainly against children and adolescents. In the first four months of that year, approximately 10,000 reports and 18,000 violations involving sexual violence—including abuse, rape, and sexual exploitation—were recorded (5). In addition, in the same year, the 17th edition of the Brazilian Public Security Forum (17ª edição do Fórum Brasileiro de Segurança Pública) revealed that 74,930 cases of rape and statutory rape were registered, the highest number ever recorded in history (6).

It is important to highlight that sexual violence is included on the list of notifiable diseases and issues in the Notifiable Diseases Information System (*Sistema de Informação de Agravos de Notificação*, SINAN) and must be reported immediately (within 24 hours and through the fastest means of communication) at the municipal level (7). This mandatory reporting allows for investigations of the issue in different groups, as shown by a time series ecological study that described the evolution of notifications of physical, sexual, and psychological violence, as well as neglect against children in Brazil between 2011 and 2019 (8). Another similar study analyzed 39,967 notifications of sexual violence and identified the temporal trend and spatial distribution of these cases among male children and adolescents, linking them to municipal development levels in Brazil (9).

Such investigations, especially on sexual violence (10–12), support the planning of adequate care for populations most vulnerable to this type of violence, help define the profile of perpetrators and main victims, contribute to identifying and addressing gaps in public policies on the subject, reduce underreporting, and assist in combating inequalities experienced by minority groups. 

Therefore, considering the magnitude, impact, and consequences of sexual violence, as well as the importance of reporting this issue to better guide control and response strategies, the present study is justified. Its objective was to analyze the temporal trend of sexual violence in Brazil between 2016 and 2022.

## Methods 

### Study design

Time series study with a quantitative approach to analyze the trend of sexual violence in Brazil from 2016 to 2022.

### Setting 

The study was conducted at the national level, considering the five geographic regions of Brazil (Central-West, North, Northeast, Southeast, and South), with data collected from notifications in the SINAN, maintained by the Ministry of Health.

### Participants 

All reported cases of sexual violence recorded in Brazil between 2016 and 2022 were included, available through the website of the Department of Informatics of the Brazilian National Health System Information Technology Department (*Departamento de Informática do Sistema Único de Saúde*, DATASUS).

### Variables 

Dependent variable (Y):

- Year of occurrence of violence (2016, 2017, 2018, 2019, 2020, 2021, and 2022).

Independent variables (X):

- Geographic regions of Brazil: Central-West, Northeast, North, Southeast, and South.
- Sociodemographic characteristics of the victim: age group (in years): 00–09, 10–19, 20–29, 30–39, 40–49, 50–59, and ≥60; sex: female, male, and ignored; race/skin color: Asian, White, Indigenous, Brown (Brazilian mixed race), Black, and ignored/blank; education level: illiterate, incomplete elementary education, complete elementary education, incomplete high school, complete high school, incomplete higher education, complete higher education, not applicable, and ignored/blank.
- Characteristics of the sexual violence event: place of occurrence: bar or similar, commercial establishment, school, collective housing, industries/construction sites, sports facility, residence, public road, other places, ignored, and blank; repeated violence: yes, no, ignored, and blank; suspected alcohol use by perpetrator: yes, no, ignored, and blank.

### Data sources and measurement

Data obtained from SINAN, publicly available through DATASUS. The variables were extracted according to the standardized fields included in the disease and issues notification forms. It should be noted that, during data collection, in the “available selections” option on the DATASUS page, specifically under the item “sexual violence,” the field “yes” was selected. 

### Bias control

The study may be subject to information bias and underreporting bias. The first refers to the incomplete completion of the notification forms; the second concerns the underreporting of cases that do not reach health services.

### Study size

The total number of records included in this analysis was 299,962 notifications of sexual violence cases in Brazil from 2016 to 2022. 

### Statistical methods

Initially, descriptive analysis was performed using Microsoft Excel, where the data were organized into tables and absolute and relative frequencies of notifications were calculated. For temporal trend analysis, Prais-Winsten linear regression was applied using Stata software, version 14.0, without exploring interactions between variables. This procedure corrected for the serial autocorrelation commonly present in time series. The annual percent change (APC) and its corresponding 95% confidence interval were calculated. The analysis of the temporal trend adopted the following criterion: a negative value of the Principal Component Analysis (PCA) indicates a decreasing trend, a positive value indicates an increasing trend, and if there is no statistical significance to the value (p-value <0.05), the trend is considered stationary. 

## Results 

Between 2016 and 2022, a total of 299,962 cases of sexual violence were reported in Brazil. The geographic regions of Brazil presented the following percentages: Southeast, 40.4%; South, 18.6%; Northeast, 17.8%; North, 13.9%; and Central-West, 9.4%. Additionally, among the reported cases of sexual violence, 88.7% (n=266,036) were female, 44.2% (n=132,489) were aged 10–19 years, 46.5% (n=139,523) identified as Brown, and 34.4% (n=103,122) had incomplete elementary education (Table 1).

**Table 1:** Absolute (n) and relative (%) distribution of the Brazilian geographic regions and the sociodemographic characteristics of the victims reported in sexual violence notifications. Brazil, 2016-2022 (n=299,962)

Variables	n (%)
Region	
Central-West	28,267 (9.4)
Northeast	53,359 (17.8)
North	41,553 (13.9)
Southeast	121,107 (40.4)
South	55,676 (18.6)
Sex	
Female	266,036 (88.7)
Male	33,866 (11.3)
Ignored	60 (0.0)
**Age group** (years)	
00–09	91,328 (30.4)
10–19	132,489 (44.2)
20–29	36,314 (12.1)
30–39	20,661 (6.9)
40–49	11,212 (3.7)
50–59	4,670 (1.6)
≥60	2,971 (1.0)
Ignored/blank	317 (0.1)
**Race/skin color**	
Asian	2,572 (0.9)
White	106,093 (35.4)
Indigenous	3,429 (1.1)
Brown	139,523 (46.5)
Black	27,614 (9.2)
Ignored/blank	20,731 (6.9)
**Education Level**	
Illiterate	2,153 (0.7)
Incomplete elementary education	103,122 (34.4)
Complete elementary education	13,550 (4.5)
Incomplete high school	24,074 (8.0)
Complete high school	22,929 (7.6)
Incomplete higher education	8,297 (2.8)
Complete higher education	6,269 (2.1)
Not applicable	62,500 (20.8)
Ignored/blank	57,068 (19.0)

An increasing trend of this type of violence was also observed at the national level (APC 8.13; 95%CI 3.38; 13.12; p-value 0.007) and in all geographic regions of Brazil: Central-West (APC 8.56; 95%CI 3.33; 14.07; p-value 0.008); Northeast (APC 10.61; 95%CI 6.60; 14.78; p-value 0.001); North (APC 5.06; 95%CI 1.09; 9.19; p-value 0.022); Southeast (APC 8.72; 95%CI 3.38; 14.36; p-value 0.008); and South (APC 7.01; 95%CI 0.41; 14.05; p-value 0.041) (Table 2).

**Table 2:** Absolute (n) and relative (%) distribution by year of notification, annual percent change (APC), 95% confidence interval (95%CI), and trend of sexual violence cases, according to the geographic regions of Brazil. Brazil, 2016-2022 (n=299,962)

Variables	2016	2017	2018	2019	2020	2021	2022	APC (95%CI)	p-value	Trend
	n (%)	n (%)	n (%)	n (%)	n (%)	n (%)	n (%)			
Brazil	31,122 (10.4)	37,379 (12.5)	41,985 (14.0)	45,878 (15.3)	39,601 (13.2)	46,991 (15.7)	57,006 (19.0)	8.13 (3.38; 13.12)	0.007	increasing
**Geographical regions**										
Central-West	2,935 (10.4)	3,512 (12.4)	3,923 (13.9)	4,228 (15.0)	3,735 (13.2)	4,290 (15.2)	5,644 (20.0)	8.56 (3.33; 14.07)	0.008	increasing
Northeast	5,135 (9.6)	6,241 (11.7)	7,215 (13.5)	7,988 (15.0)	7,088 (13.3)	9,327 (17.5)	10,365 (19.4)	10.61 (6.60; 14.78)	0.001	increasing
North	5,072 (12.2)	5,417 (13.0)	5,739 (13.8)	6,118 (14.7)	5,219 (12.6)	6,512 (15.7)	7,476 (18.0)	5.06 (1.09; 9.19)	0.022	increasing
Southeast	12,207 (10.1)	15,265 (12.6)	16,877 (13.9)	18,270 (15.1)	15,916 (13.1)	18,766 (15.5)	23,806 (19.7)	8.72 (3.38; 14.36)	0.008	increasing
South	5,773 (10.4)	6,944 (12.5)	8,231 (14.8)	9,274 (16.7)	7,643 (13.7)	8,096 (14.5)	9,715 (17.4)	7.01 (0.41; 14.05)	0.041	increasing

Regarding age group, sex, race/skin color, and education level of the victim, only four variables showed a stationary trend: ignored/blank age (APC 25.90; 95%CI -34.38; 141.59; p-value 0.405); male sex (APC 5.54; 95%CI -0.43; 11.88; p-value 0.063); ignored/blank race/skin color (APC -1.99; 95%CI -4.91; 1.01; p-value 0.147); and education level: illiterate (APC 1.68; 95%CI -2.81; 5.71; p-value 0.318). All other variables showed increasing trends (Table 3).

**Table 3:** Absolute (n) and relative (%) distribution by year of notification, annual percent change (APC), 95% Confidence Interval (95%CI), and trend of sexual violence cases, according to the victim’s age group, sex, race/skin color, and education level. Brazil, 2016-2022 (n=299,962)

Variables	2016	2017	2018	2019	2020	2021	2022	APC (95%CI)	p-value	Trend
	n (%)	n (%)	n (%)	n (%)	n (%)	n (%)	n (%)			
**Age group** (years)										
00–09	9,673 (10.6)	11,267 (12.3)	13,274 (14.5)	14,308 (15.7)	12,077 (13.2)	14,011 (15.3)	16,718 (18.3)	7.41 (2.17; 12.93)	0.014	increasing
10–19	13,734 (10.4)	16,696 (12.6)	18,525 (14.0)	19,904 (15.0)	17,192 (13.0)	21,073 (15.9)	25,365 (19.1)	8.17 (3.29; 13.28)	0.007	increasing
20–29	3,607 (9.9)	4,505 (12.4)	4,924 (13.6)	5,555 (15.3)	5,002 (13.8)	5,609 (15.4)	7,112 (19.6)	9.09 (4.59; 13.80)	0.003	increasing
30–39	2,086 (10.1)	2,571 (12.4)	2,767 (13.4)	3,157 (15.3)	2,812 (13.6)	3,203 (15.5)	4,065 (19.7)	8.79 (4.57; 13.19)	0.003	increasing
40–49	1,112 (9.9)	1,400 (12.5)	1,467 (13.1)	1,735 (15.5)	1,441 (12.9)	1,797 (16.0)	2,260 (20.2)	9.12 (4.43; 13.98)	0.004	increasing
50–59	483 (10.3)	569 (12.2)	631 (13.5)	709 (15.2)	637 (13.6)	734 (15.7)	907 (19.4)	8.79 (4.87; 12.86)	0.002	increasing
≥60	341 (11.5)	363 (12.2)	396 (13.3)	443 (14.9)	434 (14.6)	440 (14.8)	554 (18.6)	6.66 (4.53; 8.86)	<0.001	increasing
Ignored/blank	86 (27.1)	8 (2.5)	1 (0.3)	67 (21.1)	6 (1.9)	124 (39.1)	25 (7.9)	25.90 (-34.38; 141.59)	0.405	stationary
**Sex**										
Female	27,354 (10.3)	33,028 (12.4)	36,891 (13.9)	40,451 (15.2)	35,210 (13.2)	42,106 (15.8)	50,996 (19.2)	8.46 (3.85; 13.28)	0.005	increasing
Male	3,767 (11.1)	4,345 (12.8)	5,086 (15.0)	5,423 (16.0)	4,383 (12.9)	4,875 (14.4)	5,987 (17.7)	5.54 (-0.43; 11.88)	0.063	stationary
Ignored	1 (1.7)	6 (10.0)	8 (13.3)	4 (6.7)	8 (13.3)	10 (16.7)	23 (38.3)	41.15 (10.75; 79.90)	0.015	increasing
**Race/skin color**										
Asian	213 (8.3)	280 (10.9)	300 (11.7)	343 (13.3)	415 (16.1)	498 (19.4)	523 (20.3)	16.02 (14.30; 17.78)	<0.001	increasing
White	10,989 (10.4)	13,606 (12.8)	15,221 (14.3)	16,664 (15.7)	13,711 (12.9)	15,893 (15.0)	20,009 (18.9)	7.36 (1.50; 13.57)	0.023	increasing
Indigenous	345 (10.1)	401 (11.7)	442 (12.9)	544 (15.9)	421 (12.3)	550 (16.0)	726 (21.2)	9.91 (4.39; 15.74)	0.005	increasing
Brown	13,762 (9.9)	16,469 (11.8)	19,412 (13.9)	21,153 (15.2)	18,717 (13.4)	22,741 (16.3)	27,269 (19.5)	9.96 (5.20; 14.94)	0.003	increasing
Black	2,636 (9.5)	3,286 (11.9)	3,834 (13.9)	4,267 (15.5)	3,678 (13.3)	4,350 (15.8)	5,563 (20.1)	10.40 (4.65; 16.47)	0.005	increasing
Ignored/blank	3,177 (15.3)	3,337 (16.1)	2,776 (13.4)	2,907 (14.0)	2,659 (12.8)	2,959 (14.3)	2,916 (14.1)	-1.99 (-4.91; 1.01)	0.147	stationary
**Education Level**										
Illiterate	265 (12.3)	302 (14.0)	321 (14.9)	348 (16.2)	273 (12.7)	326 (15.1)	318 (14.8)	1.68 (-2.18; 5.71)	0.318	stationary
Incomplete elementary education	11,108 (10.8)	13,202 (12.8)	14,894 (14.4)	15,792 (14.4)	13,042 (12.6)	15,738 (15.3)	19,346 (18.8)	6.90 (1.34; 12.77)	0.024	increasing
Complete elementary education	1,302 (9.6)	1,613 (11.9)	1,884 (13.9)	2,085 (15.4)	1,845 (13.6)	2,155 (15.9)	2,666 (19.7)	10.27 (5.01; 15.80)	0.004	increasing
Incomplete high school	2,344 (9.7)	3,069 (12.7)	3,478 (14.4)	3,569 (14.8)	3,108 (12.9)	3,816 (15.9)	4,690 (19.5)	9.12 (2.85; 15.77)	0.013	increasing
Complete high school	1,998 (8.7)	2,670 (11.6)	2,938 (12.8)	3,494 (15.2)	3,203 (14.0)	3,753 (16.4)	4,873 (21.3)	12.65 (7.97; 17.55)	0.001	increasing
Incomplete higher education	735 (8.9)	1,004 (12.1)	1,137 (13.7)	1,364 (16.4)	1,092 (13.2)	1,220 (14.7)	1,745 (21.0)	10.83 (3.156; 19.10)	0.014	increasing
Complete higher education	450 (7.2)	663 (10.6)	770 (12.3)	954 (15.2)	967 (15.4)	1,055 (16.8)	1,410 (22.5)	17.75 (11.76; 24.08)	<0.001	increasing
Ignored	6,238 (11.0)	7,144 (12.5)	7,548 (13.2)	8,380 (14.7)	7,764 (13.6)	9,173 (16.1)	10,776 (18.9)	7.68 (4.87; 10.58)	0.001	increasing
Not applicable	6,637 (10.6)	7,712 (12.3)	9,015 (14.4)	9,892 (15.8)	8,307 (13.3)	9,755 (15.6)	11,182 (17.9)	7.21 (2.34; 12.32)	0.012	increasing

Regarding the characteristics of the sexual violence events, for place of occurrence, an increasing trend was observed for cases that occurred in: residence (APC 10.56; 95%CI 6.38; 14.91; p-value 0.001); commercial establishment (APC 10.83; 95%CI 6.49; 15.36; p-value 0.001); other places (APC 6.44; 95%CI 2.38; 10.66; p-value 0.009); and the “blank” category (APC 9.83; 95%CI 2.25; 17.97; p-value 0.020). For collective housing, schools, sports facilities, bars, and similar public places, as well as industrial/construction sites, and the ‘ignored location’ category, the trend remained stationary. Regarding repeated violence, an increasing trend was observed for all analyzed variables: yes (APC 11.34; 95%CI 6.40; 16.50; p-value 0.002); no (APC 5.71; 95%CI 0.68; 11.00; p-value 0.033); ignored (APC 6.54; 95%CI 2.46; 10.79; p-value 0.009); and blank (APC 12.61; 95%CI 1.36; 25.11; p-value 0.034). Regarding suspected alcohol use by the perpetrator, an increasing trend was also observed for all investigated variables: yes (APC 8.84; 95%CI 4.31; 13.56; p-value 0.004); no (APC 9.01; 95%CI 3.34; 15.00; p-value 0.009); ignored (APC 6.56; 95%CI 2.57; 10.74; p-value 0.008); and blank (APC 18.13; 95%CI 7.46; 29.84; p-value 0.006) (Table 4).

**Table 4:** Absolute (n) and relative (%) distribution by year of notification, annual percent change (APC), 95% confidence interval (CI95%), and trend of sexual violence cases, according to place of occurrence, whether the violence was repeated, and whether there was suspected alcohol use by the perpetrator. Brazil, 2016-2022 (n=299,962)

Variables	2016	2017	2018	2019	2020	2021	2022	APC (95%CI)	p-value	Trend
	n (%)	n (%)	n (%)	n (%)	n (%)	n (%)	n (%)			
**Place of occurrence**										
Bars or similar	343 (9.4)	440 (12.1)	499 (13.7)	561 (15.4)	393 (10.8)	487 (13.4)	913 (25.1)	10.23 (-1.15; 22.92)	0.070	stationary
Commercial establishments	327 (9.2)	439 (12.3)	466 (13.1)	535 (15.0)	473 (13.3)	612 (17.2)	714 (20.0)	10.83 (6.49; 15.36)	0.001	increasing
Schools	699 (10.7)	847 (12.9)	1,058 (16.2)	126 (619.3)	385 (5.9)	522 (8.0)	1,774 (27.1)	1.13 (-21.20; 29.80)	0.912	stationary
Collective housing	253 (9.2)	333 (12.1)	510 (18.5)	491 (17.8)	341 (12.4)	365 (13.3)	459 (16.7)	6.96 (-6.53; 22.41)	0.256	stationary
Industries/construction sites	121 (17.9)	97 (14.3)	93 (13.8)	98 (14.5)	82 (12.1)	84 (12.4)	101 (14.9)	-3.44 (-7.83; 1.14)	0.110	stationary
Sports facilities	143 (13.2)	157 (14.4)	163 (15.0)	174 (16.0)	142 (13.1)	131 (12.1)	177 (16.3)	-0.45 (-5.10; 5.28)	0.983	stationary
Residence	18,506 (9.7)	22,165 (11.6)	26,202 (13.8)	28,500 (15.0)	26,071 (13.7)	31,948 (16.8)	37,163 (19.5)	10.56 (6.38; 14.91)	0.001	increasing
Public road	4,018 (13.1)	4,746 (15.4)	4,670 (15.2)	4,715 (15.3)	3,767 (12.2)	3,999 (13.0)	4,865 (15.8)	0.21 (-5.29; 5.15)	0.923	stationary
Other places	3,187 (10.8)	3,833 (13.0)	4,000 (13.6)	4,596 (15.6)	3,949 (13.4)	4,372 (14.9)	5,490 (18.7)	6.44 (2.38; 10.66)	0.009	increasing
Ignored	3,509 (11.4)	4,304 (14.0)	4,306 (14.0)	4,919 (16.0)	3,966 (12.9)	4,447 (14.5)	5,324 (17.3)	3.90 (-0.42; 8.41)	0.068	stationary
Blank	16 (10.2)	18 (11.5)	18 (11.5)	23 (14.6)	32 (20.4)	24 (15.3)	26 (16.6)	9.83 (2.25; 17.97)	0.020	increasing
**Repeated violence**										
Yes	11,171 (9.4)	13,602 (11.4)	16,465 (13.9)	17,899 (15.1)	16,071 (13.5)	19,952 (16.8)	23,713 (19.9)	11.34 (6.40; 16.50)	0.002	increasing
No	13,293 (11.2)	15,699 (13.2)	16,952 (14.3)	18,281 (15.4)	15,312 (12.9)	17,402 (14.6)	21,898 (18.4)	5.71 (0.68; 11.00)	0.033	increasing
Ignored	6,383 (10.7)	7,718 (13.0)	8,212 (13.8)	9,356 (15.7)	7,901 (13.3)	9,162 (15.4)	10,747 (18.1)	6.54 (2.46; 10.79)	0.009	increasing
Blank	275 (9.9)	360 (13.0)	356 (12.8)	342 (12.3)	317 (11.4)	475 (17.1)	648 (23.4)	12.61 (1.36; 25.11)	0.034	increasing
**Suspected alcohol consumption by the perpetrator**										
Yes	6,406 (10.2)	7,730 (12.3)	8,706 (13.8)	9,620 (15.3)	8,635 (13.7)	9,765 (15.5)	12,117 (19.2)	8.84 (4.31; 13.56)	0.004	increasing
No	12,943 (10.0)	15,779 (12.2)	18,517 (14.3)	20,167 (15.5)	17,035 (13.1)	20,384 (15.7)	25,008 (19.3)	9.01 (3.34; 15.00)	0.009	increasing
Ignored	11,614 (11.0)	13,694 (13.0)	14,576 (13.8)	15,889 (15.1)	13,711 (13.0)	16,532 (15.7)	19,433 (18.4)	6.58 (2.57; 10.74)	0.008	increasing
Blank	159 (9.3)	176 (10.3)	186 (10.9)	202 (11.9)	220 (12.9)	310 (18.2)	448 (26.3)	18.13 (7.46; 29.84)	0.006	increasing

## Discussion 

The findings of this study suggest that sexual violence in Brazil has been persistently widespread, showing an increasing trend across all geographic regions, disproportionately affecting women, girls, adolescents, Brown individuals, and those with low education levels. The rise of this type of violence remained consistent across various settings, particularly in residences and commercial establishments. Furthermore, an increasing trend was observed in cases of sexual violence, regardless of whether the violence was repeated or involved suspected alcohol use by the perpetrator. These results demonstrate the scope and complexity of this issue, affecting different profiles and contexts.

Despite the findings, this study has limitations arising from the use of secondary data, which are subject to underreporting, incompleteness, and a lack of detail in the completion of notification forms and the SINAN, potentially compromising the accuracy needed to estimate the true magnitude of the issue. It is also important to highlight the absence of variables such as gender identity and sexual orientation, which could broaden the understanding of the phenomenon. In addition, the analyzed period coincided with the COVID-19 pandemic, which likely contributed to underreporting and to an increase in cases, due to social isolation, greater exposure of victims to perpetrators in the domestic environment, and limited access to reporting channels. However, it is essential to emphasize that these limitations do not invalidate the study nor diminish its relevance.

The increasing trend of sexual violence in Brazil can be attributed to several factors, such as the rise of sexual exploitation linked to technological advances; shortcomings in victim protection; social, cultural, and economic inequalities; and inequities in access to health care that persist in the country (9,10,13). Moreover, greater awareness among health professionals in recent years regarding situations of sexual violence may also partly explain the growth in the notification rate and, consequently, in the trend of increase of this phenomenon (8). 

Among the geographic regions of Brazil, the Southeast stood out. Notifications in this region were up to three times higher than in the Central-West, which had the lowest total number of reported cases of sexual violence in the country. Similarly, a study that analyzed the profile of sexual violence against male children in Brazil between 2009 and 2017 indicated a higher number of cases in the Southeast region (14).

This result may be related to the population density observed in Brazil. According to the latest demographic census conducted by the Brazilian Institute of Geography and Statistics (*Instituto Brasileiro de Geografia e Estatística*, IBGE), the Southeast leads in number of inhabitants, being the most populous region in the country, with approximately 85,000,000 residents, representing around 42.0% of the Brazilian population (15). Having the largest population and the most developed urban centers in Brazil, in addition to greater access to services that help victims and to resources/channels for reporting violence, may result in a higher number of notifications (16).

As already mentioned, there was an increasing trend in sexual violence across all age groups, reflecting the depth of the challenges to its prevention and control, as this reality highlights the need for change across multiple levels—cultural, political, and educational. This study also highlights the higher prevalence of this issue among individuals aged 10 to 19 years, which corroborates the findings of a similar nationwide study conducted during the COVID-19 pandemic, in which this type of violence was more prevalent among those aged 10 to 13 years (17).

Another study, conducted with adolescent girls in Brazil between 2011 and 2018, revealed that among the age groups analyzed, the 10 to 14-year-old group reported the highest number of sexual violence cases. This age group tends to be more victimized due to its greater vulnerability, as these individuals are not yet fully developed, may have limited perception, and fail to recognize themselves as victims of sexual violence, in addition to the preference of abusers for adolescents going through puberty (18). In light of this, the importance of providing sexual education to children and adolescents is reinforced to help them identify and report situations of sexual violence.

This type of violence against females showed an increasing trend, with seven times more cases recorded compared to males, whose trend remained stationary. Females are the most frequent victims of sexual offenses, as also observed in a study conducted in Maranhão between 2015 and 2020, in which more than 93.0% of the 3,142 reported cases of sexual violence were against women (19). 

Sexual violence against women is one of the most alarming manifestations of gender-based violence, reflecting a patriarchal society that is strongly oppressive and dominating, where stereotypes about the roles of men and women tend to normalize rape culture, as certain behaviors are perceived as instinctive and natural (1). Male domination that characterizes society is marked by sexism (prejudice against women based on gender) and rape culture (beliefs that encourage and justify sexual violence) (20). It is also worth noting the stationary trend of sexual violence against males, which reflects the persistence of stereotypes socially associated with masculinity. Such characteristics act as barriers to seeking professional help and may contribute to the underreporting of sexual violence in this population group.

When analyzing the reported cases of sexual violence according to the victims’ race/skin color, an increasing trend was observed across all categories, with Brown individuals showing the highest percentage of notifications. It is worth noting that, according to IBGE, more than 45.0% of the Brazilian population identifies as Brown (15), which aligns with this finding. Similarly, a study that analyzed 14,323 reports of sexual violence in Amazonas between 2012 and 2021 and conducted an epidemiological characterization of sexual violence found that Brown individuals were the most affected, accounting for approximately 78.0% of cases (21). 

The trend of sexual violence notifications among illiterate individuals remained stationary, while it was increasing across all other levels of education. This difference may be due to multiple factors, such as the barriers faced by people without education in accessing health services (22) or in understanding information about signs of sexual violence and reporting channels. Although inequity in access to healthcare remains a reality for a portion of the most vulnerable population (23), it is essential to note that the increase in notifications at other educational levels reflects the commitment and strengthening of public health services in addressing this issue. It should also be noted that the data from this study do not allow for establishing an explicit correlation between sexual violence notifications and the regional and socioeconomic distribution of the Brazilian population.

The main place of occurrence, in most of the reported cases, was the residence, consistent with findings from a similar study (11), and showing an increasing trend. This suggests that, in many cases, the home environment does not represent a truly safe place. This phenomenon may result from the proximity between perpetrator and victim, as most perpetrators are family members or individuals within the family’s social circle. Intimacy and trust may create a favorable environment for the perpetrator, making them feel comfortable committing the act of violence. In addition, homes are places with less monitoring, which facilitates the practice of violence and may give the aggressor a sense of impunity (24).

Repeated sexual violence, although less frequent than non-repeated cases, showed a growing trend. This reality corroborates the findings of a study conducted in Mato Grosso between 2013 and 2019, which sought to analyze the factors associated with the recurrence of interpersonal violence against children and adolescents (25), showing that sexual violence tends to be a recurrent issue (26). Moreover, the frequent silencing and denial of abuse represent aggravating factors for the perpetuation and recurrence of this issue (26). 

Increasing trends were also observed in notifications of non-repeated sexual violence and in places such as commercial establishments and other public spaces. These findings suggest the coexistence of different risk factors, indicating that the possibility of victimization by this type of violence remains present in public environments, especially for the most vulnerable groups. This raises a public concern, as the recurrence of sexual violence episodes in shared spaces and during daily commutes highlights the need to give greater visibility to this phenomenon, as well as to implement more effective actions for victim protection and addressing this issue (27).

A growing trend was also observed in notifications of sexual violence regardless of the suspicion of alcohol use by the perpetrator. A study conducted in Espírito Santo revealed suspected alcohol use in about 56.0% of cases (27), while another study, this time in Rondônia, found that suspected alcohol use was significantly associated with cases of sexual violence (28). Alcohol consumption is a psychoactive behavior-triggering substance that can lead to impulsive, aggressive, and uncontrolled actions and has been associated with acts of violence, including sexual violence, although it does not justify them (29).

The suspicion of non-consumption of alcohol by the perpetrator was, as already mentioned, identified in the present study. The absolute and relative frequencies over the years were examined to determine whether they remained higher than the confirmation of suspected consumption. Similarly, a study conducted in Palmas, Tocantins, which aimed to analyze reported cases of sexual violence in the municipal reference service and to characterize the profile of victims and the outcomes of health conditions, found that most perpetrators were not intoxicated (30). This finding reinforces both the patriarchal structure of Brazilian society and the fact that many perpetrators are present within the victim’s own household. 

Based on the results of this study, it can be concluded that the trend of sexual violence in Brazil has remained on the rise over the analyzed years, with broad geographic and sociodemographic distribution. This scenario highlights the deeply rooted and multifaceted nature of sexual violence, as well as the importance of strengthening epidemiological surveillance, intersectoral networks (health, public security, education, and social assistance), and ongoing education of professionals working in this network of care, focusing on early identification, support, and effective resolution. Educational campaigns in schools and community public spaces are also essential to strengthen the fight against sexual violence, to break its cycle, and to promote a culture of respect and protection for victims. To achieve this, public managers must ensure the financing, implementation, and monitoring of policies aimed at preventing sexual violence and providing care to victims. The understanding of this evidence may also serve as a basis for future research, as well as for developing strategies to ensure greater safety and protection for potential victims. 

## Data Availability

The database and analysis codes used in this study will be made available upon request from reviewers and/or the scientific community by contacting the authors at the following email address: ludiane.silva@ufpi.edu.br.
