# Supplementary material for: Temporal trend of sexual violence: time series analysis, Brazil, 2016–2022
Source: Epidemiol Serv Saude. 2026 May 1;35:e20250416. doi: 10.1590/S2237-96222026v35e20250416.en (PMC13138605; doi:10.1590/S2237-96222026v35e20250416.en)
Supplement: Translation

# Translation: Tendência temporal da violência sexual: análise de série temporal, Brasil, 2016-2022

**DOI:** 10.1590/S2237-96222026v35e20250416.pt

**Authors:** João Vitor Lima Pereira, Ludiane Rodrigues Dias Silva, Eduarda da Silva Miranda, Brenda Rainara Pereira da Silva, Izabel Cristina da Silva Carvalho, José Cláudio Garcia Lira, Adriene da Fonseca Rocha, Jéssica de Menezes Nogueira, Jardeliny Corrêa da Penha

**Affiliations:** 1 Universidade de Brasília, Programa de Residência Multiprofissional em Vigilância em Saúde, Brasília, DF, Brasil; 2 Universidade Federal do Piauí, Programa de Pós-Graduação em Saúde e Comunidade, Teresina, PI, Brasil; 3 Universidade Federal do Piauí, Coordenação do Curso de Bacharelado em Enfermagem, Floriano, PI, Brasil; 4 Universidade Federal do Piauí, Coordenação do Curso de Ciências Biológicas, Floriano, PI, Brasil

**Keywords:** Violência, Delitos Sexuais, Brasil, Monitoramento Epidemiológico, Análise de Série Temporal

## Abstract

**Objetivo:**

Analisar a tendência temporal da violência sexual no Brasil entre 2016 e 2022.

**Métodos:**

Estudo de séries temporais no qual utilizou-se os casos de violência sexual notificados, entre 2016 a 2022, no Sistema de Informação de Agravos de Notificação. Calculou-se a Variação Percentual Anual (VPA) e o seu respectivo Intervalo de confiança de 95%, utilizando-se do modelo de análise linear generalizada de Prais-Winsten.

**Resultados:**

Foram notificados 299.962 casos de violência sexual. Observou-se também tendência crescente em âmbito nacional (VPA 8,13; IC95% 3,38; 13,12; p-valor 0,007) e em todas as regiões geográficas brasileiras: Centro-Oeste (VPA 8,56; IC95% 3,33; 14,07; p-valor 0,008); Nordeste (VPA 10,61; IC95% 6,60; 14,78; p-valor 0,001); Norte (VPA 5,06; IC95% 1,09; 9,19; p-valor 0,022); Sudeste (VPA 8,72; IC95% 3,38; 14,36; p-valor 0,008); e Sul (VPA 7,01; IC95% 0,41; 14,05; p-valor 0,041). Em relação à faixa etária, ao sexo, à raça/cor da pele e à escolaridade da vítima, apenas quatro variáveis apresentaram tendência estacionária: idade ignorada/branco (VPA 25,90; IC95% -34,38; 141,59; p-valor 0,405), sexo masculino (VPA 5,54; IC95% -0,43; 11,88; p-valor 0,063), raça/cor da pele ignorada/branco (VPA -1,99; IC95% -4,91; 1,01; p-valor 0,147) e escolaridade analfabeta (VPA 1,68; IC95% -2,81; 5,71; p-valor 0,318). As demais variáveis apresentaram tendência crescente.

**Conclusão:**

A tendência de violência sexual no Brasil manteve-se ascendente, com ampla distribuição geográfica e sociodemográfica.

**Aspectos éticos**

Esta pesquisa utilizou bancos de dados de domínio público e anonimizados.:

## Introdução 

A violência sexual pode ser compreendida como qualquer ação relacionada à sexualidade de uma pessoa por meio de coerção, incluindo atos consumados, tentativas ou outras práticas forçadas (1). Ela pode ocasionar impactos diversos com repercussão, em diferentes momentos, na vida e na saúde de quem a sofre, como implicações emocionais, que afetam diretamente as relações interpessoais, afetivo-sexuais, familiares, sociais ou profissionais, seja pela perda de confiança no outro, pela insegurança ou pela forma em que se passa a enxergar o outro e a si próprio, uma vez que as vítimas carregam marcas vividas subjetivamente (2).

No contexto mundial, a violência sexual é altamente prevalente entre mulheres. A estimativa da Organização Mundial de Saúde, em 2018, foi que aproximadamente 852 milhões de mulheres com idade entre 15 e 49 anos já enfrentaram algum tipo de violência física e/ou sexual por parte de parceiros íntimos em algum momento da vida, o que configura um importante desafio para a saúde pública global e uma séria transgressão dos direitos humanos (3,4). No Brasil, a respeito dos casos notificados às autoridades policiais por meio do Disque 100 no ano de 2023, houve um aumento nas denúncias de violência sexual, principalmente contra crianças e adolescentes. Nos quatro primeiros meses desse ano, foram registradas aproximadamente 10 mil denúncias e 18 mil violações envolvendo violências sexuais, como abuso, estupro e exploração sexual (5). Ademais, no mesmo ano, a 17ª edição do Fórum Brasileiro de Segurança Pública revelou que 74.930 casos de estupro e de estupro de vulnerável foram registrados, sendo o maior número já registrado na história (6).

É pertinente destacar que a violência sexual está incluída na lista de notificação compulsória do Sistema de Informação de Agravos de Notificação e deve ser notificada imediatamente (em até 24 horas e pelo meio de comunicação mais rápido) em âmbito municipal (7). Essa obrigatoriedade da notificação permite a realização de investigações sobre o tema em diferentes grupos, como mostra um estudo ecológico de série temporal que descreveu a evolução das notificações de violência física, sexual, psicológica e de negligência contra crianças no Brasil no período de 2011 a 2019 (8). Outro estudo, de natureza semelhante, analisou 39.967 notificações de casos de violência sexual e apurou a tendência temporal e a distribuição espacial desses casos entre crianças e adolescentes do sexo masculino, relacionando-as ao nível de desenvolvimento municipal no Brasil (9).

Essas investigações, especialmente sobre a violência sexual (10-12), proporcionam o planejamento da assistência adequada ao público mais suscetível a sofrer esse tipo de violência, permitem traçar o perfil do agressor e das principais vítimas, colaboram para a identificação e o aprimoramento das lacunas nas políticas públicas voltadas à temática, reduzem os casos de violência que não chegam às autoridades competentes e colaboram para o combate às desigualdades vivenciadas por grupos minoritários. 

Dessa forma, considerando-se a magnitude, o impacto e as consequências da violência sexual, bem como a importância da notificação desse agravo, a fim de melhor direcionar as estratégias de controle e de enfrentamento, justifica-se a realização da presente pesquisa, a qual objetivou analisar a tendência temporal da violência sexual no Brasil no período de 2016 a 2022.

## Métodos 

### Delineamento do estudo

Estudo do tipo de séries temporais, de abordagem quantitativa, para analisar a tendência de violência sexual no Brasil no período de 2016 a 2022.

### Contexto 

Realizado a nível nacional, considerando-se as cinco regiões geográficas brasileiras (Centro-Oeste, Norte, Nordeste, Sudeste e Sul), com dados coletados a partir de notificações do Sistema de Informação de Agravos de Notificação (Sinan), mantido pelo Ministério da Saúde.

### Participantes 

Foram incluídas todas as notificações registradas de violência sexual no Brasil no período de 2016 a 2022, disponíveis no endereço eletrônico do Departamento de Informática do Sistema Único de Saúde (DATASUS).

### Variáveis 

Variável dependente (Y):

- Ano de ocorrência da violência (2016, 2017, 2018, 2019, 2020, 2021 e 2022).

Variáveis independentes (X):

- Regiões geográficas do Brasil: Centro-Oeste, Nordeste, Norte, Sudeste e Sul.
- Características sociodemográficas da vítima: faixa etária (em anos): 00-09, 10-19, 20-29, 30-39, 40-49, 50-59 e ≥60; sexo: feminino, masculino e ignorado; raça/cor da pele: amarela, branca, indígena, parda, preta, ignorado/em branco; escolaridade: analfabeto, fundamental incompleto, fundamental completo, médio incompleto, médio completo, superior incompleta, superior completa, não se aplica e ignorado/em branco.
- Características do evento da violência sexual: local de ocorrência: bar ou similar, comércio, escola, habitação coletiva, indústrias/construções, local de prática esportiva, residência, via pública, outros locais, ignorado e em branco; violência de repetição: sim, não, ignorado e em branco; suspeita de uso de álcool pelo agressor: sim, não, ignorado e em branco.

### Fontes de dados/Mensuração

Dados obtidos a partir do Sinan, disponíveis publicamente por meio do DATASUS. As variáveis foram extraídas de acordo com as fichas de notificação de doenças e de agravos que contêm campos padrões de preenchimento. Ressalta-se ainda que, durante a coleta de dados, na opção “seleções disponíveis” da página do DATASUS, especificamente no item “violência sexual”, o campo “sim” foi assinalado. 

### Viés 

O estudo pode apresentar viés de informação e viés de subnotificação. O primeiro, refere-se à incompletude do preenchimento das fichas de notificações; já o segundo diz respeito à sub-representação dos casos que não chegam ao serviço de saúde para serem notificados.

### Tamanho do estudo

O total de registros incluídos nesta análise foi de 299.962 notificações de casos de violência sexual no Brasil no período de 2016 a 2022. 

### Métodos estatísticos

Inicialmente, a análise descritiva foi realizada no programa Microsoft Excel, no qual se organizou os dados em tabelas e se calculou as frequências absolutas e relativas das notificações. Para a tendência temporal, aplicou-se a análise de regressão linear de Prais-Winsten no software Stata, versão 14.0, sem exploração de interações entre as variáveis. Dessa forma, corrigiu-se a autocorrelação serial comum em séries temporais. Calculou-se a Variação Percentual Anual (VPA) e o seu respectivo Intervalo de Confiança (IC) de 95%. A análise da tendência temporal adotou o seguinte critério: valor negativo da Análise de Principais Componentes indica tendência decrescente; um valor positivo, tendência crescente; e se não houver significância estatística do valor (p-valor<0,05), a tendência é estacionária. 

## Resultados 

No período de 2016 a 2022, no Brasil, foram notificados 299.962 casos de violência sexual. As regiões geográficas brasileiras apresentaram os seguintes percentuais: Sudeste, 40,4%; Sul, 18,6%; Nordeste, 17,8%; Norte, 13,9%; e Centro-Oeste, 9,4%. 

Além disso, observou-se, entre os casos notificados de violência sexual, que 88,7% (n=266.036) eram do sexo feminino, 44,2% (n=132.489) tinham entre 10 e 19 anos, 46,5% (n=139.523) consideravam-se pardos e 34,4% (n=103.122) apresentavam ensino fundamental incompleto (Tabela 1).

**Tabela 1:** Distribuição absoluta (n) e relativa (%) das regiões geográficas brasileiras e das características sociodemográficas das vítimas, contidos nas notificações de violência sexual. Brasil, 2016-2022 (n=299.962)

Variáveis	n (%)
Região	
Centro-Oeste	28.267 (9,4)
Nordeste	53.359 (17,8)
Norte	41.553 (13,9)
Sudeste	121.107 (40,4)
Sul	55.676 (18,6)
Sexo	
Feminino	266.036 (88,7)
Masculino	33.866 (11,3)
Ignorado	60 (0,0)
**Faixa etária** (anos)	
00-09	91.328 (30,4)
10-19	132.489 (44,2)
20-29	36.314 (12,1)
30-39	20.661 (6,9)
40-49	11.212 (3,7)
50-59	4.670 (1,6)
≥60	2.971 (1,0)
Ignorado/Em branco	317 (0,1)
**Raça/cor da pele**	
Amarela	2.572 (0,9)
Branca	106.093 (35,4)
Indígena	3.429 (1,1)
Parda	139.523 (46,5)
Preta	27.614 (9,2)
Ignorado/em branco	20.731 (6,9)
Escolaridade	
Analfabeto	2.153 (0,7)
Fundamental incompleto	103.122 (34,4)
Fundamental completo	13.550 (4,5)
Médio incompleto	24.074 (8,0)
Médio completo	22.929 (7,6)
Superior incompleto	8.297 (2,8)
Superior completo	6.269 (2,1)
Não se aplica	62.500 (20,8)
Ignorado/em branco	57.068 (19,0)

Notou-se também uma tendência crescente desse tipo de violência em âmbito nacional (VPA 8,13; IC95% 3,38; 13,12; p-valor 0,007) e em todas as regiões geográficas brasileiras: Centro-Oeste (VPA 8,56; IC95% 3,33; 14,07; p-valor 0,008); Nordeste (VPA 10,61; IC95% 6,60; 14,78; p-valor 0,001); Norte (VPA 5,06; IC95% 1,09; 9,19; p-valor 0,022); Sudeste (VPA 8,72; IC95% 3,38; 14,36; p-valor 0,008); e Sul (VPA 7,01; IC95% 0,41; 14,05; p-valor 0,041) (Tabela 2).

**Tabela 2:** Distribuição absoluta (n) e relativa (%) por ano de notificação, Variação Percentual Anual (VPA), Intervalo de Confiança de 95% (IC95%) e tendência dos casos de violência sexual, segundo as regiões geográficas brasileiras. Brasil, 2016-2022 (n=299.962)

Variáveis	2016	2017	2018	2019	2020	2021	2022	VPA (IC95%)	p-valor	Tendência
	n (%)	n (%)	n (%)	n (%)	n (%)	n (%)	n (%)			
Brasil	31.122 (10,4)	37.379 (12,5)	41.985 (14,0)	45.878 (15,3)	39.601 (13,2)	46.991 (15,7)	57.006 (19,0)	8,13 (3,38; 13,12)	0,007	crescente
**Regiões geográficas**										
Centro-Oeste	2.935 (10,4)	3.512 (12,4)	3.923 (13,9)	4.228 (15,0)	3.735 (13,2)	4.290 (15,2)	5.644 (20,0)	8,56 (3,33; 14,07)	0,008	crescente
Nordeste	5.135 (9,6)	6.241 (11,7)	7.215 (13,5)	7.988 (15,0)	7.088 (13,3)	9.327 (17,5)	10.365 (19,4)	10,61 (6,60; 14,78)	0,001	crescente
Norte	5.072 (12,2)	5.417 (13,0)	5.739 (13,8)	6.118 (14,7)	5.219 (12,6)	6.512 (15,7)	7.476 (18,0)	5,06 (1,09; 9,19)	0,022	crescente
Sudeste	12.207 (10,1)	15.265 (12,6)	16.877 (13,9)	18.270 (15,1)	15.916 (13,1)	18.766 (15,5)	23.806 (19,7)	8,72 (3,38; 14,36)	0,008	crescente
Sul	5.773 (10,4)	6.944 (12,5)	8.231 (14,8)	9.274 (16,7)	7.643 (13,7)	8.096 (14,5)	9.715 (17,4)	7,01 (0,41; 14,05)	0,041	crescente

Percebeu-se que, em relação à faixa etária, ao sexo, à raça/cor da pele e à escolaridade da vítima, apenas quatro variáveis apresentaram tendência estacionária, são elas: idade ignorada/em branco (VPA 25,90; IC95% -34,38; 141,59; p-valor 0,405); sexo masculino (VPA 5,54; IC95% -0,43; 11,88; p-valor 0,063); raça/cor da pele ignorada/em branco (VPA -1,99; IC95% -4,91; 1,01; p-valor 0,147); e escolaridade analfabeta (VPA 1,68; IC95% -2,81; 5,71; p-valor 0,318). As demais variáveis apresentaram tendências crescentes (Tabela 3)

**Tabela 3:** Distribuição absoluta (n) e relativa (%) por ano de notificação, Variação Percentual Anual (VPA), Intervalo de Confiança de 95% (IC95%) e tendência dos casos de violência sexual, segundo faixa etária, sexo, raça/cor da pele e escolaridade da vítima. Brasil, 2016-2022 (n=299.962)

Variáveis	2016	2017	2018	2019	2020	2021	2022	VPA (IC95%)	p-valor	Tendência
	n (%)	n (%)	n (%)	n (%)	n (%)	n (%)	n (%)			
**Faixa etária** (anos)										
00-09	9.673 (10,6)	11.267 (12,3)	13.274 (14,5)	14.308 (15,7)	12.077 (13,2)	14.011 (15,3)	16.718 (18,3)	7,41 (2,17; 12,93)	0,014	crescente
10-19	13.734 (10,4)	16.696 (12,6)	18.525 (14,0)	19.904 (15,0)	17.192 (13,0)	21.073 (15,9)	25.365 (19,1)	8,17 (3,29; 13,28)	0,007	crescente
20-29	3.607 (9,9)	4.505 (12,4)	4.924 (13,6)	5.555 (15,3)	5.002 (13,8)	5.609 (15,4)	7.112 (19,6)	9,09 (4,59; 13,80)	0,003	crescente
30-39	2.086 (10,1)	2.571 (12,4)	2.767 (13,4)	3.157 (15,3)	2.812 (13,6)	3.203 (15,5)	4.065 (19,7)	8,79 (4,57; 13,19)	0,003	crescente
40-49	1.112 (9,9)	1.400 (12,5)	1.467 (13,1)	1.735 (15,5)	1.441 (12,9)	1.797 (16,0)	2.260 (20,2)	9,12 (4,43; 13,98)	0,004	crescente
50-59	483 (10,3)	569 (12,2)	631 (13,5)	709 (15,2)	637 (13,6)	734 (15,7)	907 (19,4)	8,79 (4,87; 12,86)	0,002	crescente
≥60	341 (11,5)	363 (12,2)	396 (13,3)	443 (14,9)	434 (14,6)	440 (14,8)	554 (18,6)	6,66 (4,53; 8,86)	<0,001	crescente
Ignorada/em branco	86 (27,1)	8 (2,5)	1 (0,3)	67 (21,1)	6 (1,9)	124 (39,1)	25 (7,9)	25,90 (-34,38; 141,59)	0,405	estacionária
**Sexo**										
Feminino	27.354 (10,3)	33.028 (12,4)	36.891 (13,9)	40.451 (15,2)	35.210 (13,2)	42.106 (15,8)	50.996 (19,2)	8,46 (3,85; 13,28)	0,005	crescente
Masculino	3.767 (11,1)	4.345 (12,8)	5.086 (15,0)	5.423 (16,0)	4.383 (12,9)	4.875 (14,4)	5.987 (17,7)	5,54 (-0,43; 11,88)	0,063	estacionária
Ignorado	1 (1,7)	6 (10,0)	8 (13,3)	4 (6,7)	8 (13,3)	10 (16,7)	23 (38,3)	41,15 (10,75; 79,90)	0,015	crescente
**Raça/cor da pele**										
Amarela	213 (8,3)	280 (10,9)	300 (11,7)	343 (13,3)	415 (16,1)	498 (19,4)	523 (20,3)	16,02 (14,30; 17,78)	<0,001	crescente
Branca	10.989 (10,4)	13.606 (12,8)	15.221 (14,3)	16.664 (15,7)	13.711 (12,9)	15.893 (15,0)	20.009 (18,9)	7,36 (1,50; 13,57)	0,023	crescente
Indígena	345 (10,1)	401 (11,7)	442 (12,9)	544 (15,9)	421 (12,3)	550 (16,0)	726 (21,2)	9,91 (4,39; 15,74)	0,005	crescente
Parda	13.762 (9,9)	16.469 (11,8)	19.412 (13,9)	21.153 (15,2)	18.717 (13,4)	22.741 (16,3)	27.269 (19,5)	9,96 (5,20; 14,94)	0,003	crescente
Preta	2.636 (9,5)	3.286 (11,9)	3.834 (13,9)	4.267 (15,5)	3.678 (13,3)	4.350 (15,8)	5.563 (20,1)	10,40 (4,65; 16,47)	0,005	crescente
Ignorada/em branco	3.177 (15,3)	3.337 (16,1)	2.776 (13,4)	2.907 (14,0)	2.659 (12,8)	2.959 (14,3)	2.916 (14,1)	-1,99 (-4,91; 1,01)	0,147	estacionária
Escolaridade										
Analfabeto	265 (12,3)	302 (14,0)	321 (14,9)	348 (16,2)	273 (12,7)	326 (15,1)	318 (14,8)	1,68 (-2,18; 5,71)	0,318	estacionária
Fundamental incompleto	11.108 (10,8)	13.202 (12,8)	14.894 (14,4)	15.792 (14,4)	13.042 (12,6)	15.738 (15,3)	19.346 (18,8)	6,90 (1,34; 12,77)	0,024	crescente
Fundamental completo	1.302 (9,6)	1.613 (11,9)	1.884 (13,9)	2.085 (15,4)	1.845 (13,6)	2.155 (15,9)	2.666 (19,7)	10,27 (5,01; 15,80)	0,004	crescente
Médio incompleto	2.344 (9,7)	3.069 (12,7)	3.478 (14,4)	3.569 (14,8)	3.108 (12,9)	3.816 (15,9)	4.690 (19,5)	9,12 (2,85; 15,77)	0,013	crescente
Médio completo	1.998 (8,7)	2.670 (11,6)	2.938 (12,8)	3.494 (15,2)	3.203 (14,0)	3.753 (16,4)	4.873 (21,3)	12,65 (7,97; 17,55)	0,001	crescente
Superior incompleto	735 (8,9)	1.004 (12,1)	1.137 (13,7)	1.364 (16,4)	1.092 (13,2)	1.220 (14,7)	1.745 (21,0)	10,83 (3,156; 19,10)	0,014	crescente
Superior completo	450 (7,2)	663 (10,6)	770 (12,3)	954 (15,2)	967 (15,4)	1.055 (16,8)	1.410 (22,5)	17,75 (11,76; 24,08)	<0,001	crescente
Ignorada	6.238 (11,0)	7.144 (12,5)	7.548 (13,2)	8.380 (14,7)	7.764 (13,6)	9.173 (16,1)	10.776 (18,9)	7,68 (4,87; 10,58)	0,001	crescente
Não se aplica	6.637 (10,6)	7.712 (12,3)	9.015 (14,4)	9.892 (15,8)	8.307 (13,3)	9.755 (15,6)	11.182 (17,9)	7,21 (2,34; 12,32)	0,012	crescente

Quanto à descrição da ocorrência da violência sexual, evidenciou-se que, quanto ao local, houve tendência crescente em casos que se deram em: residência (VPA 10,56; IC95% 6,38; 14,91; p-valor 0,001); comércio (VPA 10,83; IC95% 6,49; 15,36; p-valor 0,001); outros locais (VPA 6,44; IC95% 2,38; 10,66; p-valor 0,009); e na variável “em branco” (VPA 9,83; IC95% 2,25; 17,97; p-valor 0,020). Nos locais do tipo habitação coletiva, escola, local de prática esportiva, bar ou similar, via pública, indústrias/construções e na variável de local ignorado, a tendência apresentou-se estacionária. A respeito de a violência ter sido de repetição, verificou-se tendência crescente para todas as variáveis analisadas: sim (VPA 11,34; IC95% 6,40; 16,50; p-valor 0,002); não (VPA 5,71; IC95% 0,68; 11,00; p-valor 0,033); ignorado (VPA 6,54; IC95% 2,46; 10,79; p-valor 0,009); e em branco (VPA 12,61; IC95% 1,36; 25,11; p-valor 0,034). Quanto à suspeita de uso de álcool pelo agressor, também se observou uma tendência crescente para todas as variáveis investigadas: sim (VPA 8,84; IC95% 4,31; 13,56; p-valor 0,004); não (VPA 9,01; IC95% 3,34; 15,00; p-valor 0,009); ignorado (VPA 6,56; IC95% 2,57; 10,74; p-valor 0,008); e em branco (VPA 18,13; IC95% 7,46; 29,84; p-valor 0,006) (Tabela 4).

**Tabela 4:** Distribuição absoluta (n) e relativa (%) por ano de notificação, Variação Percentual Anual (VPA), Intervalo de Confiança de 95% (IC95%) e tendência dos casos de violência sexual, segundo local de ocorrência, se foi violência de repetição e se havia suspeita de uso de álcool pelo agressor. Brasil, 2016-2022 (n=299.962)

Variáveis	2016	2017	2018	2019	2020	2021	2022	VPA (IC95%)	p-valor	Tendência
	n (%)	n (%)	n (%)	n (%)	n (%)	n (%)	n (%)			
**Local de ocorrência**										
Bar ou similar	343 (9,4)	440 (12,1)	499 (13,7)	561 (15,4)	393 (10,8)	487 (13,4)	913 (25,1)	10,23 (-1,15; 22,92)	0,070	estacionária
Comércio	327 (9,2)	439 (12,3)	466 (13,1)	535 (15,0)	473 (13,3)	612 (17,2)	714 (20,0)	10,83 (6,49; 15,36)	0,001	crescente
Escola	699 (10,7)	847 (12,9)	1.058 (16,2)	126 (619,3)	385 (5,9)	522 (8,0)	1.774 (27,1)	1,13 (-21,20; 29,80)	0,912	estacionária
Habitação coletiva	253 (9,2)	333 (12,1)	510 (18,5)	491 (17,8)	341 (12,4)	365 (13,3)	459 (16,7)	6,96 (-6,53; 22,41)	0,256	estacionária
Indústrias/construções	121 (17,9)	97 (14,3)	93 (13,8)	98 (14,5)	82 (12,1)	84 (12,4)	101 (14,9)	-3,44 (-7,83; 1,14)	0,110	estacionária
Local de prática esportiva	143 (13,2)	157 (14,4)	163 (15,0)	174 (16,0)	142 (13,1)	131 (12,1)	177 (16,3)	-0,45 (-5,10; 5,28)	0,983	estacionária
Residência	18.506 (9,7)	22.165 (11,6)	26.202 (13,8)	28.500 (15,0)	26.071 (13,7)	31.948 (16,8)	37.163 (19,5)	10,56 (6,38; 14,91)	0,001	crescente
Via pública	4.018 (13,1)	4.746 (15,4)	4.670 (15,2)	4.715 (15,3)	3.767 (12,2)	3.999 (13,0)	4.865 (15,8)	0,21 (-5,29; 5,15)	0,923	estacionária
Outros locais	3.187 (10,8)	3.833 (13,0)	4.000 (13,6)	4.596 (15,6)	3.949 (13,4)	4.372 (14,9)	5.490 (18,7)	6,44 (2,38; 10,66)	0,009	crescente
Ignorado	3.509 (11,4)	4.304 (14,0)	4.306 (14,0)	4.919 (16,0)	3.966 (12,9)	4.447 (14,5)	5.324 (17,3)	3,90 (-0,42; 8,41)	0,068	estacionária
Em branco	16 (10,2)	18 (11,5)	18 (11,5)	23 (14,6)	32 (20,4)	24 (15,3)	26 (16,6)	9,83 (2,25; 17,97)	0,020	crescente
**Violência de repetição**										
Sim	11.171 (9,4)	13.602 (11,4)	16.465 (13,9)	17.899 (15,1)	16.071 (13,5)	19.952 (16,8)	23.713 (19,9)	11,34 (6,40; 16,50)	0,002	crescente
Não	13.293 (11,2)	15.699 (13,2)	16.952 (14,3)	18.281 (15,4)	15.312 (12,9)	17.402 (14,6)	21.898 (18,4)	5,71 (0,68; 11,00)	0,033	crescente
Ignorado	6.383 (10,7)	7.718 (13,0)	8.212 (13,8)	9.356 (15,7)	7.901 (13,3)	9.162 (15,4)	10.747 (18,1)	6,54 (2,46; 10,79)	0,009	crescente
Em branco	275 (9,9)	360 (13,0)	356 (12,8)	342 (12,3)	317 (11,4)	475 (17,1)	648 (23,4)	12,61 (1,36; 25,11)	0,034	crescente
**Suspeita de uso de álcool pelo agressor**										
Sim	6.406 (10,2)	7.730 (12,3)	8.706 (13,8)	9.620 (15,3)	8.635 (13,7)	9.765 (15,5)	12.117 (19,2)	8,84 (4,31; 13,56)	0,004	crescente
Não	12.943 (10,0)	15.779 (12,2)	18.517 (14,3)	20.167 (15,5)	17.035 (13,1)	20.384 (15,7)	25.008 (19,3)	9,01 (3,34; 15,00)	0,009	crescente
Ignorado	11.614 (11,0)	13.694 (13,0)	14.576 (13,8)	15.889 (15,1)	13.711 (13,0)	16.532 (15,7)	19.433 (18,4)	6,58 (2,57; 10,74)	0,008	crescente
Em branco	159 (9,3)	176 (10,3)	186 (10,9)	202 (11,9)	220 (12,9)	310 (18,2)	448 (26,3)	18,13 (7,46; 29,84)	0,006	crescente

## Discussão 

Os achados deste estudo sugerem que a violência sexual no Brasil tem se disseminado de forma persistente, com tendência crescente em todas as regiões geográficas, afetando de forma desproporcional, principalmente, mulheres, meninas, adolescentes, pessoas pardas e com baixa escolaridade. O crescimento desse tipo de violência se manteve consistente em locais diversos, destacando-se o ambiente residencial e o comércio. Além disso, notou-se tendência crescente nos casos de violência sexual, independentemente de ser ou não de repetição ou de envolver suspeita de uso de álcool pelo agressor. Tais resultados demonstram a abrangência e a complexidade desse agravo, atingindo diferentes perfis e contextos.

Mesmo diante dos resultados obtidos, este estudo apresenta limitações decorrentes do uso de dados secundários, sujeitos a subnotificações, a incompletudes e a falta de detalhamento no preenchimento das fichas e do Sistema de Informação de Agravos de Notificação, o que pode comprometer a precisão necessária para se dimensionar a magnitude real do problema. Também se destaca a ausência de variáveis como identidade de gênero e orientação sexual, que poderiam ampliar a compreensão do fenômeno. Ademais, o período analisado coincidiu com a pandemia de covid-19, a qual, possivelmente, contribuiu para a subnotificação e para o aumento dos casos, em razão do isolamento social e da maior exposição das vítimas a agressores no ambiente doméstico, além da limitação de acesso aos canais de notificação. Porém, é fundamental destacar que essas limitações não inviabilizam o estudo nem diminuem a sua relevância.

A crescente tendência de violência sexual no Brasil pode ser atribuída a diversos fatores, como: aumento da exploração sexual em decorrência do avanço tecnológico; falhas na proteção às vítimas; desigualdades sociais, culturais e econômicas; iniquidades de acesso à saúde que persistem no país (9,10,13). Além disso, a maior sensibilização dos profissionais de saúde, nos últimos anos, frente às situações de violência sexual, também pode ser uma provável justificativa para o crescimento na taxa de notificações e, consequentemente, na tendência de aumento desse fenômeno (8). 

Dentre as regiões geográficas brasileiras, foi destaque a Sudeste. A notificação nessa região chega a ser três vezes maior do que na Centro-Oeste, região brasileira que apresentou a menor soma de notificações de violência sexual do país. De modo semelhante, uma pesquisa que buscou analisar o perfil da violência sexual contra crianças do sexo masculino no Brasil entre 2009 e 2017 indicou maior número de casos na região Sudeste do país (14).

É possível que esse resultado seja decorrente da densidade populacional expressa no Brasil. Conforme o último censo demográfico realizado pelo Instituto Brasileiro de Geografia e Estatística (IBGE), o Sudeste ocupa a liderança quanto ao número de habitantes, sendo a região mais populosa do país, com cerca de 85 milhões de residentes, o que representa, aproximadamente, 42,0% da população brasileira (15). O fato de possuir a maior população e os centros urbanos mais desenvolvidos do Brasil, além de mais acesso aos serviços que fornecem assistência às vítimas e aos recursos/canais de denúncia de violência, pode acarretar um maior número de notificações (16).

Como já mencionado, houve tendência crescente da violência sexual em todas as faixas etárias, resultado da profundidade dos fatores referentes ao seu combate, já que tal realidade aponta a necessidade de haver mudanças em várias camadas, sejam elas culturais, políticas ou educacionais. Destaca-se ainda a maior prevalência desse agravo na faixa etária de 10 a 19 anos, o que corrobora achados de pesquisa semelhante realizada, durante a pandemia de covid-19, em âmbito nacional, em que esse tipo de violência foi mais prevalente em pessoas com 10 a 13 anos de idade (17).

Outra pesquisa, realizada com mulheres adolescentes no Brasil entre 2011 e 2018, revelou que, dentre as faixas etárias avaliadas, a que mais apresentou casos de violência sexual foi a de 10 a 14 anos de idade. Essa faixa etária tende a ser mais vitimada devido a sua maior vulnerabilidade, visto que ainda não se desenvolveram completamente, podendo ter um déficit de percepção e não se enxergarem como vítimas de violência sexual, além da predileção dos abusadores por adolescentes que estejam passando pela puberdade (18). Frente a isso, reforça-se a importância de se trabalhar a educação sexual com crianças e adolescentes, a fim de ajudá-los a identificar e a denunciar situações de violência sexual.

Esse tipo de violência no sexo feminino apresentou tendência crescente, sendo registrados sete vezes mais casos do que no masculino, que manteve uma tendência estacionária. O sexo feminino é o que mais sofre delitos sexuais, conforme constatado também em pesquisa desenvolvida no Maranhão entre os anos de 2015 e 2020, na qual, de 3.142 notificações de violência sexual, mais de 93,0% foram contra mulheres (19). 

A violência sexual contra mulheres é uma das representações mais alarmantes da violência de gênero, reflexo de uma sociedade patriarcal, fortemente opressora e dominadora, pois o estereótipo sobre a função da figura masculina ou da feminina tende a normalizar a cultura do estupro, uma vez que algumas atitudes são vistas como instintivas e naturais (1). A dominação masculina que caracteriza a sociedade é marcada pelo sexismo (preconceitos contra mulheres por questão de gênero) e pela cultura do estupro (crenças que encorajam e justificam a violência sexual) (20). Convém também pontuar a tendência estacionária da violência sexual no sexo masculino, a qual reflete a persistência de estereótipos socialmente associados à masculinidade. Tal característica atua como barreira na busca por ajuda profissional, podendo contribuir para a subnotificação da violência sexual nesse grupo populacional.

Analisando-se as notificações dos casos de violência sexual segundo a raça/cor da pele das vítimas, notou-se tendência crescente em todas as variáveis dessa categoria, destacando-se a raça/cor da pele parda com maior percentual de notificações. Vale ressaltar que, de acordo com o IBGE, mais de 45,0% da população brasileira considera-se parda (15), o que vai ao encontro do presente achado. Assim como neste estudo, uma pesquisa que analisou 14.323 notificações da violência sexual no Amazonas entre os anos de 2012 e 2021 e realizou uma caracterização epidemiológica da violência sexual encontrou que pessoas pardas foram as que mais sofreram desse agravo, totalizando aproximadamente 78,0% dos casos (21). 

A tendência de notificações de violência sexual entre pessoas analfabetas se manteve estacionária, enquanto nos demais níveis de escolaridade foi crescente. Essa diferença decorre de múltiplos fatores, como barreiras enfrentadas pelas pessoas sem escolaridade para acessarem os serviços de saúde (22) ou para compreenderem informações referentes aos sinais da violência sexual e aos seus canais de denúncia. Apesar da iniquidade de acesso à saúde ainda ser uma realidade para uma parcela da população mais vulnerabilizada (23), é pertinente destacar que o aumento nas notificações nos demais níveis educacionais reflete o comprometimento e o fortalecimento dos serviços públicos de saúde no enfrentamento desse agravo. Ressalta-se também que os dados desta pesquisa não possibilitam o estabelecimento de correlação explícita entre as notificações de violência e a distribuição regional e socioeconômica da população brasileira.

O principal local de ocorrência, na maioria dos casos notificados, foi a residência, em concordância com os resultados obtidos em estudo semelhante (11), e em tendência crescente. O que traz a ideia de que, muitas vezes, o ambiente domiciliar não representa um local realmente seguro. Esse fenômeno pode ser consequência da proximidade entre o agressor e a vítima, uma vez que a maioria dos violentadores são familiares, ou pessoas que pertencem ao círculo de convívio da família. A intimidade e a confiança podem ocasionar um ambiente favorável ao agressor, fazendo com que ele se sinta à vontade para cometer a violência. Além disso, as residências são locais com menos monitorização, o que facilita a prática de violência e pode dar ao agressor uma sensação de impunidade (24).

A violência sexual de repetição, embora menos frequente que a não repetida, mostrou tendência de crescimento. Tal realidade corrobora os achados de um estudo realizado no Mato Grosso no período entre 2013 a 2019, que buscou analisar os fatores associados à reincidência da violência interpessoal contra crianças e adolescentes (25), evidenciando que a violência sexual tende a ser um agravo de repetição (26). Ademais, o frequente silenciamento e a negação do abuso representam fatores agravantes para a perpetuação e a reincidência desse agravo (26). 

Verificaram-se ainda tendências crescentes de notificações de violência sexual não repetida e em locais como estabelecimentos comerciais e outros espaços públicos. Esses achados sugerem uma coexistência de diferentes fatores de risco, apontando que permanece a possibilidade de vitimização para violência dessa natureza em ambientes públicos, especialmente para os grupos mais vulnerabilizados. Isso desperta um alerta de ordem pública, pois a recorrência de episódios de violência sexual em locais de uso coletivo e durante os deslocamentos cotidianos ressalta a necessidade de maior visibilidade para esse fenômeno, bem como a implementação de ações mais eficazes de proteção às vítimas e de enfrentamento dessa problemática (27).

Também foi observada uma tendência crescente de notificações de violência sexual independentemente da suspeita de uso de álcool pelo agressor. Um estudo realizado no Espírito Santo revelou que houve suspeita de uso de álcool em cerca de 56,0% dos casos (27), enquanto outro estudo, dessa vez em Rondônia, apontou que a suspeita de uso de álcool foi significativamente relacionada aos casos de violência sexual (28). O consumo de álcool, por se tratar de uma substância psicoativa que pode ocasionar comportamentos impulsivos, agressivos e descontrolados, tem sido associado a atos de violência, incluindo a sexual, embora não os justifiquem (29).

A suspeita de não uso de álcool pelo agressor foi algo, como já mencionado, identificado na presente pesquisa, e as frequências absolutas e relativas ao longo dos anos foram investigadas quanto a se manterem maiores que a confirmação da suspeita. De modo semelhante, uma pesquisa desenvolvida em Palmas, Tocantins, que objetivou analisar os casos de violência sexual notificados no serviço de referência do município e caracterizar o perfil das vítimas e o desfecho dos agravos, constatou que a maior parte dos agressores não estava alcoolizada (30), o que ratifica o patriarcado existente na sociedade brasileira e o fato de o agressor estar na própria residência da vítima. 

Com os resultados desta pesquisa, conclui-se que a tendência de violência sexual no Brasil manteve-se ascendente ao longo dos anos analisados, com ampla distribuição geográfica e sociodemográfica. Essa conjuntura ressalta o caráter enraizado e multifacetado da violência sexual, além da importância no fortalecimento da vigilância epidemiológica, da articulação da rede intersetorial (saúde, segurança pública, educação e assistência social) e da educação permanente dos profissionais que atuam nessa rede de atenção com enfoque na identificação precoce, no acolhimento e na resolutividade. Campanhas educativas em escolas e em espaços públicos comunitários também são primordiais para fortalecer a luta contra a violência sexual e romper o seu ciclo, além de promover a cultura do respeito e a proteção às vítimas. Para isso, os gestores públicos precisam garantir financiamento, implementação e fiscalização das políticas voltadas à prevenção da violência sexual e à atenção às vítimas. A compreensão dessas evidências também poderá servir de embasamento para futuras pesquisas, bem como para traçar estratégias para proporcionar mais segurança e proteção às potenciais vítimas. 

## Data Availability

O banco de dados e os códigos de análise utilizados na pesquisa serão disponibilizados sob demanda dos pareceristas e/ou da comunidade científica, mediante contato com os autores através do seguinte e-mail: ludiane.silva@ufpi.edu.br.
